# Successful re-treatment of a relapsed V600E mutated HCL patient with low-dose vemurafenib

**DOI:** 10.18632/oncoscience.111

**Published:** 2014-12-26

**Authors:** Caroline Bailleux, Guillaume Robert, Clémence Ginet, Daniel Re, Antoine Thyss, Isabelle Sudaka, Isabelle Peyrottes, Paul Hofman, Patrick Auberger, Frederic Peyrade

**Affiliations:** ^1^ Department of Medical Oncology, Centre Antoine Lacassagne, Nice, France; ^2^ INSERM, U1065, TEAM 2, Nice, France; ^3^ University of Nice, Nice, France; ^4^ Laboratory of Hematology, CHU Nice, Nice, France; ^5^ Department of Pathology, Centre Antoine Lacassagne, Nice, France; ^6^ Laboratory of Clinical and Experimental Pathology, Pasteur Hospital, CHU Nice

**Keywords:** hairy-cell leukemia, V600E mutation, relapse, vemurafenib, re-treatment

## Abstract

Hairy cell leukemia (HCL) is a chronic B-cell lymphoproliferative disorder that accounts for 2% of all leukemia. Recent identification of the recurrent V600E BRAF mutation in a majority of HCL patients has led some teams to evaluate the clinical potential of vemurafenib, a BRAF V600 specific inhibitor in a limited number of refractory HCL patients. Recently, we published the case of an HCL patient successfully treated with a low dose of vemurafenib. Eight months after the ending of treatment this patient relapsed. We present here the successful retreatment of this patient with a second line of vemurafenib. Our data suggest for the first time that vemurafenib at the dose of 240 mg once a day could be sufficient to maintain a complete hematological remission after an initial induction treatment with low-dose vemurafenib (2 × 240 mg) daily without inducing major toxicity.

## INTRODUCTION

Hairy cell leukemia (HCL) is a rare chronic B-cell lymphoproliferative disorder that predominantly affects middle-aged males (sex ratio 1/4). HCL is characterized by the occurence of CD103-positive circulating B cells, pancytopenia and splenomegaly [1]. In 2011, *Tiacci et al.* discovered that 100 % of HCL patients harbor a mutation at codon 600 of BRAF (V600E) leading to a constitutive activation of the BRAF kinase and of all downstream kinases [2]. This genetic event seems therefore to have an important role in the pathogenesis of HCL. *Dietrich et al.* reported in 2012 a case of refractory HCL treated with increasing doses of vemurafenib an ATP-competitive BRAF V600 inhibitor that has been shown to have potent antitumor activity in BRAF V600 mutated melanomas [3]. This drug was shown to exhibit remarkable activity at high doses (480 mg 4x/day) on both splenomegaly and blood counts. Prolonged remission at 6 months after treatment has been documented [4]. Recently, we presented the case of a refractory HCL patient treated with low dose of vemurafenib (240 mg 2x/day) and leading to complete remission [5]. However, after a period of treatment suspension this patient has relapsed. Here, we present a successful re-treatment schema of a relapsed V600E mutated HCL with low-dose vemurafenib.

## RESULTS

**Figure 1:**
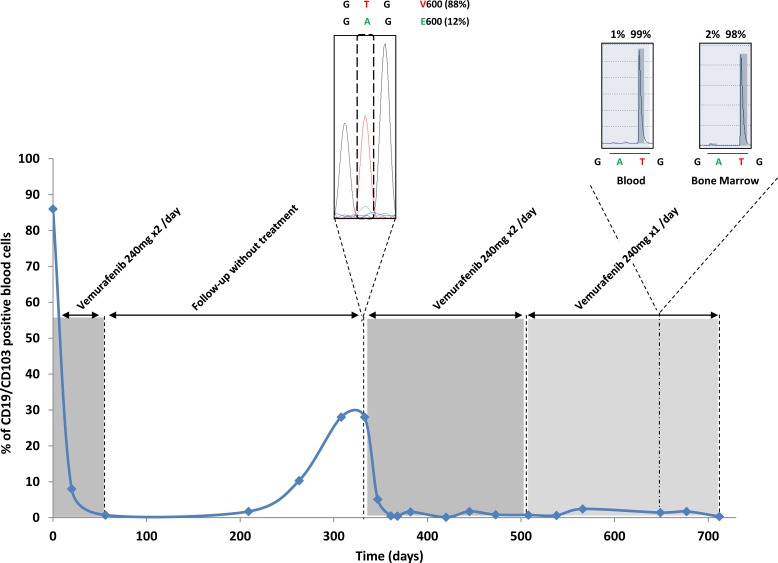
Monitoring of leukemic markers in peripheral blood Monitoring of CD19/CD103 positive cells in peripheral blood, during the first vemurafenib treatment at 240 mg x2/day (from Day 0 to Day 56), eight months before relapse (from Day 57 to Day 332), during vemurafenib re-treatment at 240 mg x2/day (from Day 333 to Day 507) and after treatment decrease to 240 mg x1/day (from Day 508 to Day 712). The identification of the BRAF V600E mutation was performed at Day 333 by Sanger sequencing in blood leukemia cells and at Day 649 by pyrosequencing in peripheral blood samples and bone marrow aspiration. The results are expressed as the percentages of mutant BRAF (V600E) alleles versus total peripheral white blood cells.

In March 2013, seven months after completion of low-dose vemurafenib treatment (240 mg 2x/day) of an HCL patient, increased levels of peripheral CD19/CD103 double positive HCL cells (10.3%) were detected and further confirmed at Day 308 (28%). Decrease of hemoglobin level was also documented at day 301 (11.2 g/dl), whereas patient remained asymptomatic (Table. 1). The presence of 12% BRAF V600E mutation was established by Sanger sequencing in blood leukemia cells at day 308 corresponding to 28% of CD19/CD103 double positive HCL cells (Figure. 1). Considering the efficiency and tolerance of the previous treatment, vemurafenib was then reintroduced at day 333 at an initial dose of 240 mg twice daily. Samples for performing the monitoring of leukemic cells were thus taken more frequently to observe the evolution and impact of vemurafenib re-treatment. The CD19/CD103 positive cell fraction decreased from 28.0% to 5.1% at day 347 (only 2 weeks after the reintroduction of vemurafenib) and to 0.5% at day 361 (4 weeks after the reintroduction of vemurafenib) (Figure. 1). Taking into account the early relapse after seven months and the short course vemurafenib treatment (8 weeks), we decided to maintain the vemurafenib treatment at 240 mg twice a day for 20 supplemental weeks. At day 473, the blood cell counts were clearly improved with no cytopenia detectable. At this time the hemoglobin level was 13.9 g/dl without transfusion and the leukocytes and platelets counts were 5.7 × 109/L and 372 × 109/L, respectively.

**Table 1:** Evolution of the peripheral blood cell counts (Leukocytes, Platelets, Neutrophils), Hb levels and double staining of CD19+/CD103+ cells), during the first vemurafenib treatment at 240 mg x2/day (from Day 0 to Day 56), eight months before relapse (from Day 57 to Day 332), during vemurafenib re-treatment at 240 mg x2/day (from Day 333 to Day 507) and after treatment decrease to 240 mg x1/day (from Day 508 to Day 712).

Time(days)	0	20	56	90	207	263	301	308	333	341	347	361	382	420	473	508	538	566	594	649	677	712
Treatment	240 mg 2x/day			follow-up without treatment					240 mg 2x/day							240 mg lu/day						
Leukocytes(10^9^/L)	14.4	1.8	3.2	5.4	3.9	4.4	4.7	-	-	4.3	2.6	2.6	3.6	5.4	5.7	6.2	8.0	7.6	6.1	8.2	8	7.5
Hb (g/dl)	6.9	7.9	9.2	12.6	12.6	12.1	11.2	-	-	9.0	9.1	9.2	9.9	12.5	13.9	13.7	13.9	14.2	13.5	10.4	9.5	13.6
Platelets (10^9^/L)	24.0	31.0	253.0	473.0	205.0	284.0	195.0	-	-	99.0	134.0	241.0	288.0	286.0	372.0	310.0	330.0	310.0	319.0	375.0	462.0	298.0
Neutrophils (10^9^/L)	2.0	1.0	2.4	4.1	2.3	1.8	2.4	-	-	1.3	1.6	1.5	2.9	4.2	4.4	4.8	6.2	6.2	4.5	6.6	6.2	5.8
CD19^+^/CDl03^+^(%)	86.0	8.0	0.7	0.8	1.7	10.3	-	28.0	28.0	-	5.05	0.46	1.64	0.1	0.8	0.65	0.55	2.39	-	1.4	1.66	0.3

Nevertheless, the patient presented a weight loss of 4 Kg corresponding to 6% of his body mass at day 508, that is, 200 days after re-treatment with vemurafenib at 240 mg twice a day. This weight loss is not unusual and has been already observed in melanoma patients treated with this drug.

In the absence of peripheral CD19/CD103 double positive HCL cells and considering the durable improvement of the blood cell counts, we decided to decrease the dose of vemurafenib to 240 mg once a day to improve tolerance and to obtain a weight gain (Day 508). Accordingly, we also introduced nutritional care with protein supplements. After one month, we obtained a stable weight and after four months a weight gain of 4 Kg (Day 649). On that date, the patient also still showed a complete hematological response (normal hematologic blood counts and no evidence of HCL cells in peripheral blood smear) (Table. 1). The percentage of BRAF (V600E) alleles was 1% and 2% in peripheral blood and bone marrow samples respectively (Figure. 1). These values are comparable to those of healthy volunteer donors and are compatible with the total absence of cells carrying the V600E mutation of BRAF in this patient. In conclusion, we showed here that a prolonged treatment with vemurafenib at 240 mg once a day is sufficient to maintain a normal hemogram for at least 20 weeks and to prevent the relapse in patient.

**Figure 2:**
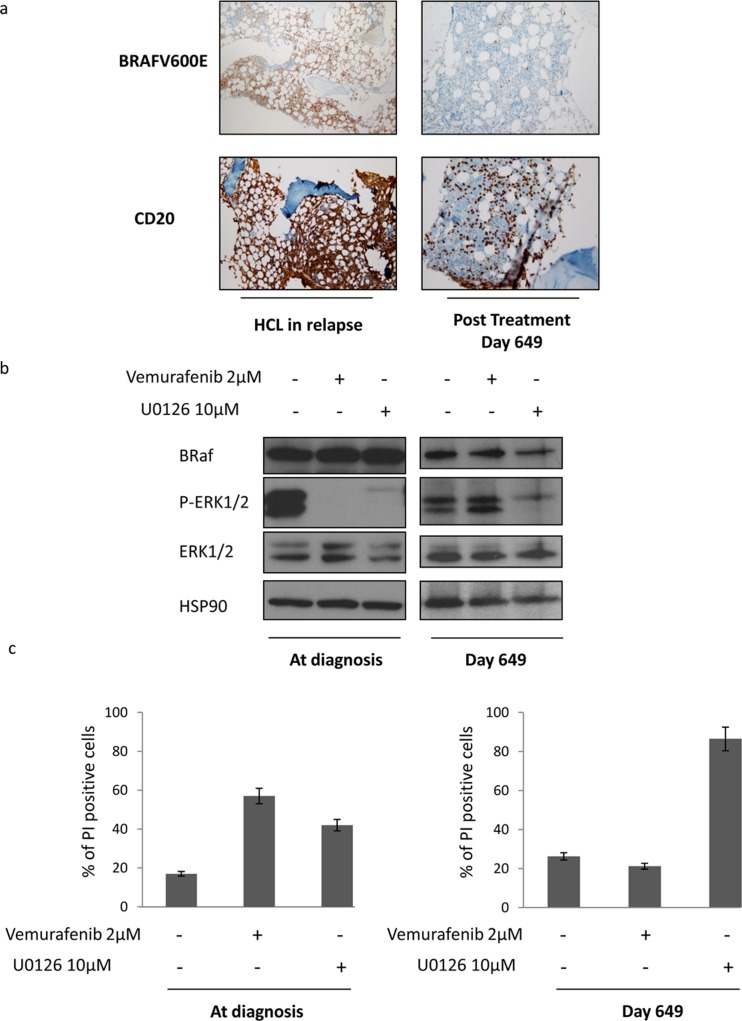
Vemurafenib reduces the number of HCL in bone marrow and leads to a decrease of MAPK pathway activation a. Bone marrow biopsies performed during a full-blown relapse diagnosis and at Day 649 was stained with a mutated anti-BRAF allele antibody and anti-CD20 antibody. b. At Diagnosis (Day 0) and at Day 649 white blood cells from blood samples were treated with vemurafenib 2 μM or U0126 10 μM. Twenty four hours later, whole-cell lysates were prepared, and expression of BRaf, P-ERK1/2, ERK1/2 and HSP90 was visualized by western blotting. c. Cells were treated as described in b., stained with Propidium Iodide and cell death was analyzed by flow cytometry.

We also studied the bone marrow response in this patient. At day 649, corresponding to eleven months after the second treatment with vemurafenib and six months after dose reduction to 240 mg/day, flow cytometry analysis detected 1.4% of CD19/CD103 positive blood cells. Only 2% of BRAF (V600E) alleles were detected by pyrosequencing (Figure. 1). A mutated anti-BRAF antibody staining was performed on bone marrow that revealed the absence of residual disease on bone marrow slides as compared to the same HCL patient when he relapsed (Figure. 2a). The patient thus presented a minimal residual disease (MRD) on bone marrow biopsy lower than the one observed after the first treatment with vemurafenib, with no evidence of morphological disease and a complete hematologic remission.

We finally analyzed the status of activation of MAP kinases downstream of RAF during treatment with vemurafenib. At diagnosis (Day 0) we observed that the MAP kinase pathway was upregulated in HCL cells (86% of PBMC in blood) as shown by a sustained level of ERK1/2 phosphorylation (Figure. 2b left panel). Inhibition of the mutated form of BRAF with vemurafenib triggered a quick and complete dephosphorylation of the downstream kinase ERK1/2 and also induced a strong increase of HCL cell death (Figure. 2c left panel).

Of note, vemurafenib did not inhibit ERK1/2 phosphorylation downstream of BRAF neither in cells collected from the blood sample at day 649 (Figure. 2b right panel). Accordingly, vemurafenib failed to kill patient's cells in identical conditions (Figure. 2c right panel). These data confirm the absence of BRAF V600E positive cells at day 649 characterized previously by pyrosequencing. Finally, we used a MEK specific inhibitor (U0126) to test the capacity to inhibit the ERK pathway downstream of BRAF and to induce cell death in normal and pathological conditions. We observed that both at time of diagnosis and at day 649, this inhibitor strongly inhibited the MAPK pathway and significantly increased cell death (Figure. 2b and 2c).

## DISCUSSION

Recent identification of the recurrent V600E BRAF mutation in a huge majority of HCL patients has led some teams to evaluate the clinical potential of vemurafenib a BRAF V600 specific inhibitor. A first trial reported the successful treatment of an HCL patient with high doses of vemurafenib (1920 mg/day) [6]. We recently described that a low dose of vemurafenib (480 mg/day) was as effective as the higher dose to treat an HCl patient at an advanced stage of his disease [5].

We describe here, that vemurafenib treatment suspension leads to a relapse of this HCL patient after seven month. We first demonstrated the necessity to follow very regularly HCL patients under vemurafenib treatment but also to monitor the persistence of HCL cells even though a negative BRAF V600E pyrosequencing is detected in peripheral blood and bone marrow. We also showed that vemurafenib re-treatment of HCL patient experiencing a relapse after a first round of vemurafenib is highly efficient to induce death of leukemic cells and a quick normalization of patient's blood formulation. Taking into account the previous relapse after vemurafenib withdrawall, we decided to maintain a daily treatment with 240 mg 2x/day. Despite the low dose of vemurafenib used, we observed a significant weight loss in our patient, an adverse drug event already reported in clinical trials on melanoma [7]. Our findings illustrate the necessity of lowering the dose of vemurafenib after a first period of reduction in tumor burden at higher dose.

In conclusion, 2 years approximately after the management of our HCL patient we propose a therapeutic regimen including a first period of induction with vemurafenib at 240 mg 2x/day, followed by a consolidation/maintenance with vemurafenib at 240 mg 1x/day. In addition, throughout the management of the patient, it appears important to perform hemograms and to monitor evolution of leukemic cells every week during the induction phase and every month during the phase of maintenance. It now seems clear that vemurafenib is a treatment of choice for patients suffering HCL that are refractory to conventional chemotherapies including cladribine, pentostatin, rituximab or alpha interferon. Two independent clinical trials for large scale evaluation of the benefit of vemurafenib in refractory HCL patients are currently ongoing in the US (Clinical trial: NCT01711632) and Europe (eudract_number:2011-005487-13) that should extend and confirm the results obtained in isolated patients. Moreover, it would be also important to evaluate the impact of this treatment in first line after diagnosis of a Hairy Cell Leukemia.

## METHODS

### Primary Cells Isolation

Blood samples from the HCL patient were collected from the Oncology Department of the Centre Antoine Lacassagne, Nice. Informed consent was obtained according to institutional guidelines. Mononuclear cells were isolated from blood samples by density centrifugation (Ficoll-PaqueTM Plus), washed 3 times with PBS, then red blood cells were lysed in Ammonium chloride buffer and finally Mononuclear cells were resuspended in PBS 2mM EDTA 5% BSA.

### Bone marrow biopsy

Double staining flow cytometry with CD19+/CD103+ and BRAF V600E pyrosequencing was performed on bone marrow aspiration coming from iliac crest. Immunohistochemical staining was performed on dewaxed paraffin sections with the Ventana Benchmark XT automated slide stainer using anti-CD20 primary antibody (clone L26, Ventana), and anti-Human BRAF V600E Monoclonal Antibody (Clone VE1) was purchased from Spring Bioscience (Pleasanton, CA, USA).

### Reagents and antibodies

Sodium fluoride and orthovanadate, phenylmethylsulfonyl fluoride, aprotinin and leupeptin were purchased from Sigma (Saint-Louis, MO, USA). Anti-ERK1/2, anti-phospho-ERK1/2 and HRP conjugated anti-rabbit antibodies were from Cell Signaling Technology (Beverly, MA, USA). Anti-Hsp90 antibody was purchased from Santa Cruz Biotechnology (Santa Cruz, CA, USA). Anti-CD19 and anti-CD103 conjugated antibodies were purchased from miltenyi (Bergisch Gladbach, Germany). HRP-conjugated anti-mouse antibody was from Dakopatts (Glostrup, Denmark).

### Measurement of cell death

After stimulation, HCL cells were stained with propidium iodide. Then, staining cells were analyzed by flow cytometry.

### Western blot

After stimulation with vemurafenib or U0126, cells were lysed at 4°C in lysis buffer. Lysates were centrifuged at 10 000g for 10 min at 4°C and supernatants were supplemented with concentrated SDS sample buffer. A total of 30 μg of protein were separated on 12% polyacrylamide gel and transferred onto polyvinylidene difluoride (PVDF) membrane (Immobilon-P, Millipore, Bedford, MA, USA). After blocking non-specific binding sites, the membranes were incubated with specific antibodies, washed three times and finally incubated with HRP-conjugated antibody for 1 h at room temperature. Immunoblots were revealed using the enhanced chemiluminescence detection kit (Amersham Biosciences, Uppsala, Sweden).

### Pyrosequencing

Quantitative estimation of BRAF V600E mutants was performed with the *therascreen*® BRAF Pyro Kit (Qiagen). Briefly, PCR amplification of *region flanking* amino acid 600 of BRAF was performed on a PTC-200 thermal cycler (MJ Research, Waltham, MA). 1 μl of each isolated DNA was analyzed per run. Pyrosequencing was performed on the PyroMark Q24 platform (Qiagen) using the PyroMark Gold Q24 reagents. Pyrograms were generated with the PyroMark Q24 software (v. 2.0.6.) and data were analyzed manually or with a plug-in tool provided by Qiagen. Sequences surrounding the site of interest served as normalization and reference peaks for quantification and quality control. Dispensation order was as follows: 5′-GCT ACT GTA GCT AGT ACG AAC TCA-3′. Two different “sequence to analyze” were used: 5′-YAY TGT AGC TAG ACS AAA AYC ACC -3′ or 5′-CHC TGT AGC TAG ACS AAA ATY ACC -3′ for manual analysis. Samples with 5% mutated alleles or more were scored as mutation positive.

### Sequencing

Genomic DNA of the patient was extracted and subjected to PCR with the following primers (Forward : 5′-TCATAATGCTTGCTCTGATAGGA-3′ – Reverse : 5′-GGCCAAAAATTTAATCAGTGGA-3′). Finally, the amplified DNA from exon 15 was sent to be sequenced by *GATC*-Biotech (Konstanz, Germany).

### Percentage of mutated cells

The percentage of mutated alleles was measured in PBMC by Sanger sequencing at day 333 by determining the proportion of T and A nucleosides at position 1799 of the BRAF gene. At day 645, the quantification was performed on both blood and bone marrow samples using pyrosequencing, which quantitatively detects the proportion of T and A nucleosides at position 1799 of the BRAF gene. It is noteworthy that this percentage reflects the number of mutated alleles. Since the V600E mutation is monoallelic, the percentage of mutated cells will be two times higher than the percentage of the mutated alleles.
